# Exploring links between Chinese military recruits' psychological stress and coping style from the person-environment fit perspective: The chain mediating effect of self-efficacy and social support

**DOI:** 10.3389/fpsyg.2022.996865

**Published:** 2022-11-03

**Authors:** Chao Wu, Guangdong Hou, Yawei Lin, Zhen Sa, Jiaran Yan, Xinyan Zhang, Ying Liang, Kejian Yang, Yuhai Zhang, Hongjuan Lang

**Affiliations:** ^1^Department of Nursing, Fourth Military Medical University, Xi'an, China; ^2^Department of Urology, Xijing Hospital, Fourth Military Medical University, Xi'an, China; ^3^69245 Troops of the Chinese People's Liberation Army, Xinjiang, China; ^4^Department of Engineer, Army 75 Group Military Hospital, Kunming, China; ^5^69243 Troops of the Chinese People's Liberation Army, Xinjiang, China; ^6^The 960th Hospital of the PLA Joint Logistics Support Force, Jinan, China; ^7^Department of Health Statistics, School of Preventive Medicine, Fourth Military Medical University, Xi'an, China

**Keywords:** psychological stress, coping style, self-efficacy, social support, Chinese recruits

## Abstract

The choice of coping style of recruits under psychological stress in the process of military task execution has been an important topic in the promotion of military operations and cohesion of military forces. Taking a positive coping style under psychological stress can help recruits overcome the negative effects of stress and improve military morale and group combat effectiveness. Although soldiers' psychological stress in the process of military mission execution having an impact on coping style has been studied by a large body of literature, very little literature has focused on the mechanism of self-efficacy and social support between recruits' psychological stress and coping style from the person-environment fit perspective. Therefore, this study was conducted to analyze the impact of recruits' psychological stress on coping style through a chain mediation model and to discuss the role of self-efficacy and social support in this relationship. Two waves of survey data were utilized to test the research hypotheses on a sample of 1028 Chinese recruits performing military tasks. The results indicated that recruits' psychological stress negatively impacted positive coping styles and positively correlated with negative ones. In addition, self-efficacy and social support mediated the relationship between psychological stress and positive coping style, and self-efficacy mediated the relationship between psychological stress and negative coping style. More importantly, self-efficacy and social support play the chain mediating effect between psychological stress and positive coping style.

## Introduction

Along with the advancement of the new military revolution, modern military tasks are high intensity, high standards, and tough environments, requiring soldiers to act with a high degree of unity (Reger et al., 2018; MacGregor et al., 2020). Under such circumstances, they face various external stimuli and produce different degrees of psychological stress reactions (Friedl, 2018; Kang et al., 2020). Psychological stress is a state of physical and mental tension that arises when an individual perceives a change in the external environment and recognizes the threat or challenge posed by the environmental change in order to cope with the change (Bernstein, 2016; Claes, 2018). Research showed that patients with higher total scores on affective dysregulated temperaments were more likely to have higher hopelessness which was an important predictor of suicidality (Serafini et al., 2012). Psychological stress will bring many negative effects, and how to reduce psychological stress has become a research hotspot (Hopper et al., 2019; Umbrello et al., 2019). Physical exercise is regarded as an effective way to solve this problem (Belvederi Murri et al., 2018; Izquierdo-Alventosa et al., 2020). However, there are some common misperceptions regarding the treatment of psychological stress and the effectiveness in treating core features of psychological stress is generally underappreciated (Belvederi Murri et al., 2018).

Military psychological stress is a specific emotional state that arises in stimulating and stressful environments. It is a physiological and psychological response of military personnel through their cognitive evaluation of the environment and form of military activities. Military psychological stress is closely related to the physical and mental health of military personnel and the performance of military tasks. It also can affect the adoption of strategies and the choice of coping styles during their tasks (Cunitz et al., 2019; Lin et al., 2020). Psychological stress is a common problem in all militaries, and this problem is especially prominent among recruits (Johnson et al., 2018). Recruits are an important supplement to the combat effectiveness of the army which is also largely determined by their performance in the military task. However, they are particularly prone to psychological stress due to factors such as not adapting to a new environment, being far from home, and difficulty in loading military tasks. The prevalence of psychological stress among recruits in the U.S. Army is 28.2%, 33.1% among recruits in the Brazilian Army, and 31.5% in Chinese Army (Jackson et al., 2011). Studies have shown that individuals in a state of psychological stress influence the choice of coping styles and action strategies, and there is a significant correlation between psychological stress and coping styles (Liu et al., 2020; Zhao et al., 2021). Coping styles are the emotional and behavioral styles that individuals exhibit in response to various dilemmas (Zhao, 2021). In military missions, there are various unpredictable stressful stimuli, and the stress from these stimuli can cause mental tension in soldiers and negatively affect their physical and mental health. In this way, the stress can affect the choice of different coping styles, which in turn affects the effectiveness of military missions (Romero et al., 2020; Zhao et al., 2020). Adopting a negative coping style can increase soldiers' mental stress or mental tension, solve problems negatively and passively, which tends to immerse individuals in negative emotions, reduce coping ability, and harm individual health status, as well as being detrimental to the advancement of military tasks and the improvement of troop combat effectiveness (Zhang et al., 2017). As an important supplement and important component of the force, guiding recruits to adopt a positive coping style will keep them in a good emotional state, which is important for them to quickly adapt to the force, maintain recruits' physical and mental health, and ensure the completion of military tasks (Niebuhr et al., 2013; Burbage et al., 2021). Therefore, it is important to understand the recruits' choice of coping styles and pathways of action in psychological stress situations during military missions.

However, research on recruits' coping styles during military missions is not yet mature and well developed, let alone how psychological stress influences coping styles. The currently recognized factors that influence the coping style are attributed to individual factors and external factors. Individual factors refer to age (Stevenson et al., 2012), personal alexithymia (Shen and Xing, 2020), personality (Jurczak et al., 2019), psychological stress (Zhao et al., 2021), etc. External factors include stress (Ryu et al., 2020), social support (Yu et al., 2020), and family environment (Zhang et al., 2020). The current studies focus on the influence of individual or external factors on coping style and do not consider their joint or even interactive effects. Our research is expected to provide reference for alleviating the psychological stress of recruits and promoting them to choose positive coping styles.

## Research theory and hypothesis

The Person-Environment Fit Theory (PEFT) is a theory commonly applied in management and psychology (Arias et al., 2020; Wang D. et al., 2021). PEFT refers to the mutual function between an individual and the environment when an individual makes a certain behavior (Wang and Wang, 2018). Fit is a two-way function, with person-environment fit referring to the supply-need balance between an individual and the environment. And individual-environment interaction factors should be taken into account to effectively explain an individual's behavior in their environment. Through this mutual function, analyses and discussions between the individual and the environment could help to clarify the real relationship between the two factors. The discussion of person-environment fit could avoid errors caused by separating the individual or environment considerations (Xiao et al., 2021).

In previous studies on the impact of psychological stress on coping styles, PEFT was not taken into account (Zhou et al., 2017; Ding et al., 2021; Zhao et al., 2021). In our study, both individual and environmental factors should be applied to replace the single trait theory in order to clarify the relationship between psychological stress and coping style. Although prior studies discussed the concept of PEFT in regard to multitasking research (Liu et al., 2019; Bowe, 2020). We do not know the mechanism behind the association between psychological stress and coping styles, especially in the soldiers. So, it is the motivation of our study to apply PEFT to explore the path of psychological stress on coping styles and help soldiers choose positive coping styles when they are subjected to psychological stress when performing major tasks. Therefore, from the perspective of positive psychology and within the framework of PEFT, our research will look for individual and environmental variables related to psychological stress and copying styles in the following sections, and assume and verify their chain mediating role.

When performing military tasks, recruits are unfamiliar with military tasks and are in a state of high physical and mental workload, thus making them prone to stress (Yu et al., 2016). Psychological stress is closely related to task performance and psychological health of military personnel, especially strong acute psychological stress is likely to cause military personnel to adopt negative coping strategies, leading to failure of military tasks and causing serious and lasting psychological trauma to military personnel (Hatzimanolis et al., 2018). Studies have shown that individuals in a state of psychological stress can affect the choice of coping styles, which can lead individuals to choose negative coping styles to cope (Wang and Wang, 2019; Fu et al., 2020). Therefore, this study proposes the following research hypothesis based on this.

### Hypothesis 1: Recruits' psychological stress is negatively related to positive coping styles

Secondly, PEFT points out that individual factors are an important aspect in making a certain behavior. We choose self-efficiency as the individual factor. Self-efficacy refers to an individual's subjective judgment of whether he or she can successfully perform an achievement behavior, and also refers to people's confidence or belief in their ability to achieve the behavioral goals in a particular domain, and is an individual's belief that he or she can succeed. Good self-efficacy is beneficial for increasing individuals' psychological resilience (Zhang et al., 2021) and alleviating psychological stress levels (Shahrour and Dardas, 2020; Cano et al., 2021). Self-efficacy is regarded as an important factor to alleviate psychological stress (Hendrix et al., 2016; Indelicato et al., 2017). It can buffer psychological stress well and the better self-efficacy is more likely to exhibit the more positive behaviors, such as self-management (Nott et al., 2021), better job performance (Downes et al., 2021), and a positive coping style (Wang and Zhang, 2021; Wu et al., 2021). Therefore, individuals with good self-efficacy tend to adopt a positive coping style in the process of event handling (Lu et al., 2021). Given that self-efficacy is an important determinant of the choice of positive coping style, it is feasible to hypothesize that recruits' psychological stress has a negative effect on self-efficacy, and self-efficacy has a positive effect on positive coping style and a negative effect on negative coping style.

### Hypothesis 2: Self-efficacy significantly mediates the relationship between psychological stress and coping styles

Thirdly, the main logic of PEFT shows that the environmental is another important factor. Social support means that individuals can feel, perceive or receive care or assistance from others (Milette et al., 2020; Wang Y. et al., 2021). Social support can help individuals get comfort and overcome negative emotions (Eidelman et al., 2019; Gaffey et al., 2020). The reason why we choose social support as the environmental factor is that social support can alleviate individual psychological stress, eliminate individual psychological barriers, and promote individual mental health. Soldiers are a special social group, shouldering the important task of safeguarding national interests and defending the people (Gagnon and Reilly, 2021). They live in a closed camp, living together and facing the same arduous training tasks (McDonald et al., 2019). In this situation, external social support, especially organizational support, is particularly important (Sipos et al., 2019; Karstoft et al., 2021). A meta-analysis of American soldiers shows that social support can effectively reduce the occurrence of posttraumatic stress disorder (Bourassa et al., 2020; Blais et al., 2021). And research shows that social support can alleviate psychological stress through care and counseling (Falak et al., 2020; Gallagher et al., 2021a). At the same time, social support and coping style are also closely related. The higher the degree of social support perceived by individuals, the more inclined they are to adopt positive coping styles (Li et al., 2021). Therefore, we propose the following research hypothesis:

### Hypothesis 3: Social support significantly mediates the relationship between recruits' psychological stress and coping styles

Fourthly, the researches on self-efficacy and social support show that they are positively correlated (Wu and Sheng, 2019; Al-Dwaikat et al., 2021). Individuals with high self-efficacy can actively seek social support in the face of difficulties. At the same time, individuals with higher external social support can usually benefit more from self-efficacy (Iwanowicz-Palus et al., 2021; Miller et al., 2021). It is further found that when the individual's self-efficacy improves, it is easier to perceive social support (Shoji et al., 2014). Therefore, we propose the following research hypothesis:

### Hypothesis 4: Recruits' self-efficacy is positively correlated with social support

Finally, according to PEFT, the individual-environment interaction factors should be taken into account to effectively explain an individual's behavior in their environment. Because self-efficacy and social support are closely related to psychological stress and coping style, in the process of choosing the coping style of soldiers under psychological stress, we choose self-efficacy as an individual factor and social support as an environmental factor. We speculate that when soldiers suffer from psychological stress, self-efficacy can help them seek and better perceive social support, which can help them choose to adopt positive coping styles. Self-efficacy and social support, as two important individual and environmental factors, may plays crucial mediating role in the relationship between psychological stress and coping styles. Therefore, based on this thesis this study proposes the following research hypothesis:

### Hypothesis 5: Self-efficacy and social support play the role of chain intermediary in the relationship between recruits' psychological stress and coping styles

**Figure 1:**
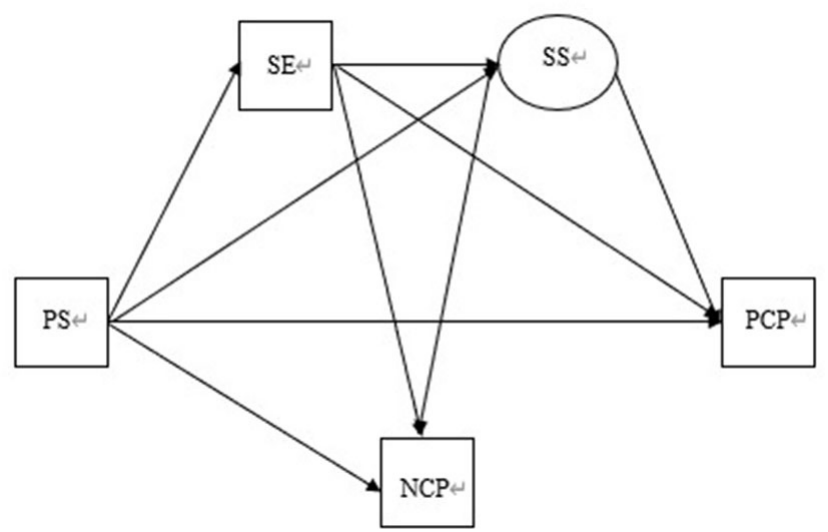
The multiple mediators of psychological stress and coping style hypothesized: (1) Soldiers' psychological stress will affect coping style. (2) Self-efficacy significantly mediates the relationship between psychological stress and coping style. (3) Self-efficacy is positively related to social support. (4) Social support significantly mediates the relationship between psychological stress and coping style. (5) Self-efficacy and social support play the role of chain intermediary in the relationship between psychological stress and coping style. PS, psychological stress; SE, Self-efficacy; SS, social support; PCP, positive coping style; NCP, negative coping style.

The above assumptions are shown in Figure 1. However, the above influences and mechanisms of action of self-efficacy, social support, and psychological stress on coping styles are at a conjectural and hypothetical stage, lacking corresponding systematic sorting and research. Therefore, this study aims to explore the influence of self-efficacy, social support, and psychological stress on the coping styles of Chinese recruits on military missions and their mechanisms of action based on PEFT in combination with previous studies, hoping to explore the reasons affecting their coping styles in-depth, helping them make positive choices and adopt positive coping styles in the heavy military tasks and under the state of psychological stress.

## Method

### Participants

Since recruits are trained collectively, and in order to save research time and funds, a convenient sampling method was used to select 1,100 recruits of a Chinese Army unit performing a major task to conduct a survey, excluding those who had recently experienced major life events. The inclusion criteria were PLA soldiers with < 1 year of service. The exclusion criteria were those who were not willing to participate or were absent during the survey. A total of 1,100 data were collected for this study during this phase. Among the 1,100 questionnaires, 1,059 were valid questionnaires. Two weeks later 1,059 were asked to complete the questionnaires concerning social support and coping styles, and a total of 1,028 valid data were collected. The effective recovery rate of the questionnaire was 93.45%.

### Measures

#### Psychological stress

Psychological stress was measured by the Psychological Stress Self-Evaluation Test (PSET) (Lin et al., 2021), which is an effective tool to evaluate the degree of psychological stress response of soldiers in both peacetime and wartime. There are 10 items in the PSET. A three-level scoring method is adopted to reflect the recruits' physiological, psychological and behavioral reactions under stress. The higher the score, the higher the degree of psychological stress. Cronbach's alphas coefficient is 0.811.

#### Coping styles

Coping styles were measured by the Simplified Coping Style Questionnaire (SCSQ) (Cheng et al., 2021). The scale is used to evaluate the coping styles of individuals. It has a total of 20 items, which is composed of two factors: positive coping style and negative coping style. It adopts the 4-level scoring method. The Cronbach's alphas coefficient of each dimension is 0.749 and 0.821.

#### Self-efficacy

Self-efficacy was measured by the General Perceived Self-Efficacy Scale (GSES) (Caruso et al., 2016). This scale is used to evaluate the individual's confidence in completing something. There are 10 items in total. It adopts the 4-level scoring method. The higher the score, the stronger the sense of self-efficacy. Cronbach's alphas coefficient is 0.884.

#### Social support

Social support was measured by the Perceived Social Support Scale (PSSS) (Karaer and Akdemir, 2019). The scale emphasizes individual self-understanding and self-perceived social support. There are 12 items in total and includes three dimensions: objective support, subjective support and utilization of support. The evaluation is conducted according to the 7-level scoring method. Cronbach's alphas coefficient is 0.928. Cronbach's alphas coefficients of each dimension vary from 0.812 to 0.883.

### Data collection

The group measurement is conducted by professionals with unified guidelines and filled in anonymously. Before filling in the questionnaire, the examiner explained the purpose and requirements of the survey in detail and asked the participants to answer truthfully when they understood the items on the scale and participants could opt-out of participation in the study at any time if they didn't want to participate in the research. The questionnaires were filled in and collected on the spot and the filling time shall be controlled at about half an hour. Because our study used cross-sectional data analysis, we contacted soldiers for data collection at two-time points to avoid the common method bias in the study data. In the first stage, the paper questionnaires on demographic variables, psychological stress and self-efficacy were distributed to the soldiers with the promise that all information would be kept strictly confidential.

### Data analysis

To test the research hypotheses of this study, the following process was conducted. Firstly, the factors were included as latent variables in structural equation modeling (SEM) analysis to test for common method bias. Secondly, the hypothesized models were tested for validity by Mplus8.0, which was chosen for the analysis because it provides high-quality path analysis to help researchers visualize the relationships and effects of the variables, and the mathematical method used by Mplus8.0 is also one of the most effective and reliable methods. In addition, the hypothesized model was tested for the significance of mediating effects by the Bootstrap method (2,000 replicate draws), and *P* < 0.05 was established as statistically significant.

## Results

### Common method deviation test

In this study, the questionnaire is filled in by self-reporting, so it may cause the problem of common method deviation (Voloh et al., 2020). Exploratory factor analysis was performed on all items of the four questionnaires. We found that the first common factor interpretation rate was 34.75%, which was less than the critical standard of 40% (Lash et al., 2021). This showed the common method bias couldn't be a concern in this study.

### Demographic characteristics and the comparison of coping styles

With the help of the commander of the army, 1,028 out of 1,100 recruits completed our survey. Among them, 14 recruits dropped out of the survey, 51 gave incomplete surveys, with the effective response rate of 94.05%. The average age of the final sample was 19.05 years (*SD* = 1.64). The other demographic characteristics of the recruits are presented in Table 1.

**Table 1:** Demographic characteristics of recruits and the comparison of coping styles (*N* = 1,028).

**Variable**	**Category**	**N (%)**	**PCS**	**NCS**
Place of residence	City	526(51.17)	37.83 ± 5.45	2.57 ± 0.97
	Countryside	502(48.83)	36.43 ± 6.25	2.44 ± 1.01
*t*			3.823	2.140
*P*			< 0.001	0.033
Whether or not the only-children	Yes	436(42.41)	37.52 ± 5.64	2.57 ± 0.99
	No	592(57.59)	36.86 ± 6.06	2.46 ± 0.99
*t*			1.776	1.750
*P*			0.076	0.080
Education levels	Junior high school	310(30.16)	37.63 ± 5.84	15.16 ± 4.26
	Senior high school	116(11.28)	34.41 ± 6.32	13.31 ± 3.40
	Technical secondary school	192(18.68)	35.78 ± 6.70	13.20 ± 3.71
	Undergraduate	410(39.88)	38.19 ± 4.99	15.02 ± 3.92
*F*			17.551	15.844
*P*			< 0.001	< 0.001
Adaptation to troops	Not adapted	20(1.95)	32.00 ± 6.66	18.20 ± 4.63
	Quite adapted	421(40.95)	36.57 ± 5.45	15.94 ± 3.86
	Very adapted	587(57.10)	37.73 ± 6.06	13.40 ± 3.71
*F*			12.863	64.739
*P*			< 0.001	< 0.001
Health condition	Sub-health	102(9.92)	35.42 ± 5.57	2.66 ± 0.94
	Health	926(90.08)	37.33 ± 5.90	2.49 ± 0.99
*t*			−3.121	1.623
*P*			0.002	0.105
Number of hobbies	None	27(2.63)	33.11 ± 9.04	16.37 ± 4.44
	1~2	505(49.12)	36.21 ± 5.54	14.62 ± 3.92
	>2	496(48.25)	38.32 ± 5.77	14.33 ± 4.08
*F*			23.520	3.572
*P*			< 0.001	0.028
Family monthly income (RMB)	< 1,000	75(7.30)	36.32 ± 6.98	13.41 ± 3.30
	1,000~3,000	401(39.01)	36.26 ± 6.08	14.15 ± 3.90
	3,001~5,000	347(33.75)	37.19 ± 5.59	14.43 ± 3.83
	>5,000	205(19.94)	39.08 ± 5.12	15.85 ± 4.51
*F*			11.153	10.859
*P*			< 0.001	< 0.001

### Descriptive analysis of psychological stress, self-efficacy, social support and coping styles scores

As shown in Table 2, the psychological stress score of recruits in this study was (14.01 ± 3.08). The score of self-efficacy was (26.98 ± 5.72). The score of social support was (68.01 ± 11.42), and (23.1 ± 4.21) for objective support, (22.81 ± 4.21) for subjective support, (22.04 ± 4.22) for support utilization. The score of positive coping style was (37.14 ± 5.89), and for negative coping style was (14.53 ± 4.02).

**Table 2:** The scores of psychological stress, self-efficacy, social support and coping styles.

**Scales and dimensions**	**Number of items**	**Minimum**	**Maxium**	**Average score**	**Score**
Psychological stress	10	10	30	1.40 ± 0.31	14.01 ± 3.08
Self-efficacy	10	10	40	2.70 ± 0.57	26.98 ± 5.72
Social support	12	13	84	5.67 ± 0.95	68.01 ± 11.42
Objective support	4	4	28	5.79 ± 1.05	23.15 ± 4.21
Subjective support	4	4	28	5.70 ± 1.05	22.81 ± 4.21
Support utilization	4	4	28	5.51 ± 1.06	22.04 ± 4.22
Positive coping style	12	12	48	3.10 ± 0.49	37.14 ± 5.89
Negative coping style	8	8	32	1.82 ± 0.50	14.53 ± 4.02

### Correlations among study variables

In Table 3, recruits' psychological stress clearly showed a negative correlation with self-efficacy (*r* = −0.184, *p* < 0.001), social support (*r* = −0.163, *p* < 0.001) and positive coping style (*r* = −0.142, *p* < 0.001) and showed a positive correlation with negative coping style (*r* = 0.415, *p* < 0.001). Self-efficacy was positively correlated with social support (*r* = 0.414, *p* < 0.001) and positive coping style (*r* = 0.417, *p* < 0.001).

**Table 3:** The correlation between psychological stress, self-efficacy, social support and coping styles.

		**1**	**2**	**3**	**4**	**5**	**6**	**7**
1	Psychological stress							
2	Self-efficacy	−0.184^**^						
3	Social support	−0.163^**^	0.414^**^					
4	Objective support	−0.124^**^	0.363^**^	0.893^**^				
5	Subjective support	−0.129^**^	0.391^**^	0.914^**^	0.725^**^			
6	Support utilization	−0.187^**^	0.369^**^	0.903^**^	0.695^**^	0.753^**^		
7	Positive coping style	−0.142^**^	0.417^**^	0.447^**^	0.377^**^	0.424^**^	0.411^**^	
8	Negative coping style	0.415^**^	−0.009	−0.023	−0.006	−0.006	−0.062^*^	0.107^*^

* *p* < 0.05,

** *p* < 0.01.

### Chain intermediary role

Structural equation modeling has requirement for sample size. When the number of entries is too large, more parameters need to be estimated by SEM. If the sample size is small, large parameter bias may be produced by using the original items. Item parceling can improve the communalities and reduce random error, which is an effective method to solve this problem. For the Perceived Social Support Scale, we packaged them according to its respective dimensions. Then we tested whether the fitted index of the constructed structural equation model conformed to the requirements. The results show that the fit indexes are χ^2^ = 38.719, *df* = 8, χ^2^/*df* = 4.840, Tucker-Lewis Index (TLI) = 0.966, Comparative Fit Index (CFI)=0.987, Root Mean Square Error of Approximation (RMSEA) = 0.060, 90% CI: 0.043–0.080, Standardized Root Mean Square Residual (SRMR) = 0.022.

**Figure 2:**
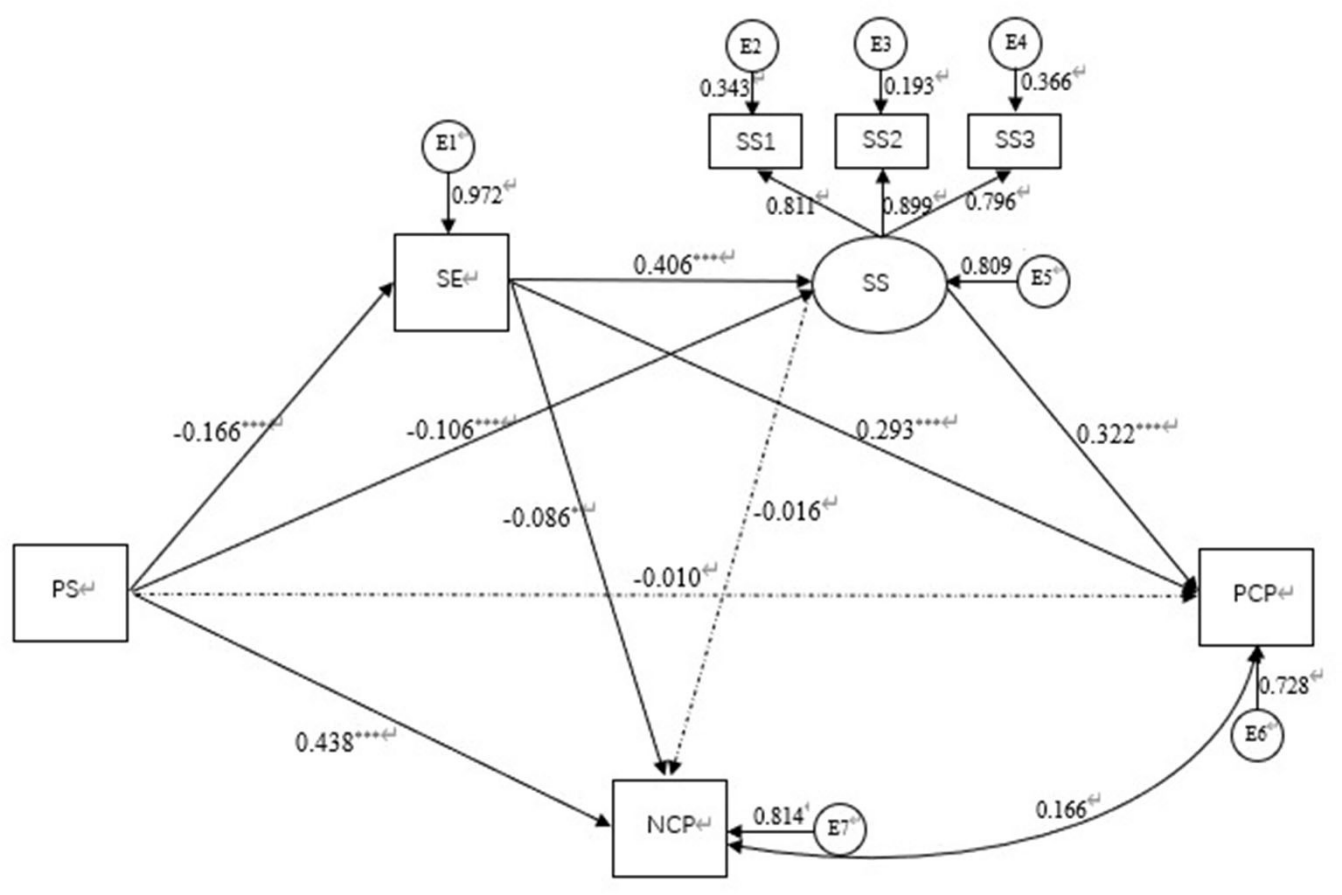
Standardized relationship path diagram. PS, psychological stress; SE, self-efficacy; SS, social support; SS1, objective support; SS2, subjective support; SS3, support utilization; PCP, positive coping style; NCP, negative coping style. : *p < 0.05, ***p < 0.001.

Figure 2 depicts the mediating effect model. Psychological stress indirectly affects positive coping style through self-efficacy (β = −0.049, SE = 0.011, *p* = 0.000) and social support (β = −0.034, SE = 0.012, *p* = 0.000). Self–efficacy and social support play the total chain mediating effect between psychological stress and positive coping style (β = −0.022, SE = 0.007, *p* = 0.002, R^2^ = 0.272).

Psychological stress had a significant positive impact on negative coping style (β = 0.438, SE = 0.032, *p* = 0.000), and indirectly affected negative coping style through self–efficacy (β = −0.014, SE = 0.007, *p* = 0.029). Other paths were not significant. The indirect effect of self–efficacy on psychological stress and negative coping style accounted for 3.88% of the total effect of psychological stress on negative coping style (total effect = 0.421, total indirect effect = −0.017, *p* = 0.023, R^2^ = 0.186).

Table 4 showed the confidence interval of mediating effect value in chain mediated model. The results showed that the path from psychological stress to positive coping style through self–efficacy and social support respectively were significant. Confidence intervals were 95% CI [−0.070, −0.031] and [−0.057, −0.015] which didn't contain 0, indicating that the mediating effects were significant. And the path from psychological stress to positive coping style through the chains of self–efficacy and social was significant and played the role of a complete chain intermediary. Confidence intervals were 95% CI [−0.036, −0.010]. The path from psychological stress to negative coping style through self–efficacy was significant. Confidence intervals were 95% CI [−0.027, −0.001]. However, the other paths were not significant. The total mediating effect value of the model of from psychological stress to positive coping style was −0.105, *p* < 0.01 and the 95 % CI [−0.143, −0.066] didn't contain 0, indicating that the total mediating effect was significant. The total mediating effect value of the model of from psychological stress to negative coping style was −0.017, *p* = 0.023 and the 95 % CI [−0.032, −0.002] didn't contain 0, indicating that the total mediating effect was significant. The specific path model diagram was shown in Figure 2.

**Table 4:** Confidence interval of mediating effect value in chain mediated model (2,000 bootstrap samples).

**Model path**	**Estimate**	**95% CI**	
		**LLCI**	**ULCI**
PS SE PCP	−0.049	−0.070	−0.031
PS SS PCP	−0.034	−0.057	−0.015
PS SE SS PCP	−0.022	−0.036	−0.010
PS SE NCP	−0.014	−0.027	−0.001

## Discussion

Our study found that self-efficacy and social support mediated the relationship between psychological stress and positive coping style, and self-efficacy mediated the relationship between psychological stress and negative coping style. More importantly, self-efficacy and social support play the chain mediating effect between psychological stress and positive coping style. And our results highlighted the importance of self-efficacy and social support as interventional factors to improve the physical and mental health and combat effectiveness of recruits. With the development of China's military, troops continue to expand their tasks, especially in exercises, field training, combat duty, and other major military tasks where there is much competitive assessment and difficult environmental conditions, the overload and high intensity training of soldiers is gradually increasing, which is a severe test of physical and psychological endurance for them (Hua et al., 2018; Chu et al., 2021). Because new recruits are new to the military and don't adapt to the military environment, the recruit training period is a time when young soldiers feel high occupational stress and are prone to develop psychological stress, which adversely affects their physical and mental health and also leads to their low enthusiasm for training and lower training performance (Guo et al., 2019; Wang et al., 2019). Under such circumstances, the choice of their coping style is related to the army's combat effectiveness, especially in psychological warfare plays a decisive and dominant role. Therefore, efforts to improve their psychological stress coping ability and coping style selection can help improve military mission execution effectiveness (Tao et al., 2020).

The purpose of this study is to explore how self-efficacy and social support influence recruits' coping style selection under psychological stress state with the guidance of PEFT, to reinforce the significance of self-efficacy and social support on recruits' positive coping style selection as well as to help them select positive coping styles under psychological stress state. What's more, this study aims to help them overcome passive, and retreating coping styles, and it is critical to help them to improve physical and mental health and the combat effectiveness of troops.

In this study, we found that in terms of positive coping styles, there were differences in positive coping style scores among recruits with different places of residence, adaptation to the army, different health status, number of hobbies, and different family income levels (*p* < 0.01). In terms of negative coping styles, there were differences in negative coping style scores among recruits with different places of residence, adaptation to the army, number of hobbies, and different family income levels (*p* < 0.05). Self-efficacy and social support played a fully chained mediating role in the selection of positive coping styles under psychological stress. During military tasks, recruits suffering from psychological stress could seek better social support by exerting their own self-efficacy and select positive coping styles to cope with the difficulties of the moment through the combined effect of internal and external factors. Therefore, self-efficacy and social support are what we need to highlight in the process of selecting coping styles for recruits suffering from psychological stress.

It was found that there was a negative correlation between self-efficacy and psychological stress levels (Liu et al., 2020). High self-efficacy recruits may match self-beliefs and expectations with military environment better. When faced with tough training life, they cope with the difficulty optimistically, and stimulate a sense of self-wort. In this way, self-efficacy is an important positive factor in reducing psychological stress reactions (Molero Jurado et al., 2019; Belanger et al., 2020). The level of self-efficacy can have an impact on how they perceive stress and how to cope with it. Related studies in the U.S. military have found that military self-efficacy moderates the relationship between stressful stimuli and tension. Compared with those with low self-efficacy, high self-efficacy individuals have the less negative effects on physiological stress (Wald et al., 2016). Also, individuals with high self-efficacy are more likely to adopt a positive coping style when choosing the copying style. Therefore, health management departments and soldiers should care about the mental health and self-efficacy of recruits, stimulate the training enthusiasm of recruits with high self-efficacy, and set training participants with clear goals and timely assessments. What's more, they should promote recruits' self-efficacy gradually, build a good interpersonal atmosphere. It will help them build self-confidence, feel the value of their existence, and enhance their self-efficacy. The above methods can also be promoted among ordinary people.

When individuals are under mental stress, individuals tend to adopt a negative and passive coping style, which tends to make individuals reduce their coping ability and fail to adapt to the stressful environment actively. The conflicts between cognition and behavior may occur, which damages individuals' physical and mental health. Furthermore, it may exacerbate burnout or stress (John-Henderson and Ginty, 2020; Gallagher et al., 2021b). If recruits are able to receive more social support from their loved ones, comrades, and organizations in coping with stressful situations. It is beneficial to improve recruits' strategies and abilities to relieve stressful situations and to adopt positive coping strategies in the process of coping style selection (Perry et al., 2021). Research has shown that when the social support provided by an individual's surrounding environment, they will take the more positive and proactive coping style and the intensity of perceived stress will relatively decrease (Thompson et al., 2016). Since recruits perform special tasks in the military environment, it is important to give full play to organizational support. Through encouragement and help from peers in the group and timely psychological guidance from the troops, recruits will overcome negative emotions, alleviate psychological stress and face training tasks positively.

To sum up, we should pay attention to the special group of recruits and highlight their psychological conditions and improve their self-efficacy and strengthen the degree of social support for them. What's more, the necessary psychological guidance and psychological support are also important. In this way, we can help them maintain physical and mental health, and overcome psychological stress. They will choose positive coping methods to cope with the difficulties in military training tasks and improve the combat effectiveness of the troops.

## Limitation

There are still some limitations of this study. First, we selected both internal and external environmental factors to explore a behavior according to PEFT. It is clear that there are more the two variables involved in this study that could affect the choice of coping styles of recruits, which is a part of future research that could go deeper. Second, this study used self-reported cross-sectional data. Given the retrospective nature of the present study, the prospective study is needed in the future research and to further reduce the impact of common method bias, future studies should attempt to conduct longitudinal follow-up data to verify the reliability of the existing findings. Moreover, this study is a cross-sectional study design, which can't carry out a causal link between various variables.

## Data availability statement

The original contributions presented in the study are included in the article/supplementary material, further inquiries can be directed to the corresponding author.

## Ethics statement

Ethical review and approval was not required for the study on human participants in accordance with the local legislation and institutional requirements. Written informed consent from the patients/ participants or patients/participants legal guardian/next of kin was not required to participate in this study in accordance with the national legislation and the institutional requirements.

## Author contributions

Design of the study: CW, KY, and YLin. Acquisition of data: HL, ZS, and YLia. Analysis and reporting of data: YZ. Drafting the manuscript: CW and XZ. All authors contributed to the article and approved the submitted version.

## Conflict of interest

The authors declare that the research was conducted in the absence of any commercial or financial relationships that could be construed as a potential conflict of interest.

## Publisher's note

All claims expressed in this article are solely those of the authors and do not necessarily represent those of their affiliated organizations, or those of the publisher, the editors and the reviewers. Any product that may be evaluated in this article, or claim that may be made by its manufacturer, is not guaranteed or endorsed by the publisher.
